# Iron-Dependent Cell Death: A New Treatment Approach against Pancreatic Ductal Adenocarcinoma

**DOI:** 10.3390/ijms241914979

**Published:** 2023-10-07

**Authors:** Carlos Lopez-Blazquez, Carlos Lacalle-Gonzalez, Lara Sanz-Criado, Michael Ochieng’ Otieno, Jesus Garcia-Foncillas, Javier Martinez-Useros

**Affiliations:** 1 Translational Oncology Division, OncoHealth Institute, Health Research Institute—Fundación Jimenéz Diaz, Fundación Jimenéz Díaz University Hospital/Universidad Autónoma de Madrid (IIS-FJD/UAM), 28040 Madrid, Spain; carlos.lblazquez@gmail.com (C.L.-B.); lara.sanz@quironsalud.es (L.S.-C.);; 2 Department of Medical Oncology, Fundación Jiménez Díaz University Hospital, 28040 Madrid, Spain; carlos.lacalle@quironsalud.es; 3 Area of Physiology, Department of Basic Health Sciences, Faculty of Health Sciences, Rey Juan Carlos University, 28922 Madrid, Spain

**Keywords:** pancreatic ductal carcinoma, reactive oxygen species, ferroptosis, apoptosis, antioxidants, oxidants, FOLFIRINOX, arsenic

## Abstract

Pancreatic ductal adenocarcinoma (PDAC) is a devastating tumor type where a very high proportion of people diagnosed end up dying from cancer. Surgical resection is an option for only about 20% of patients, where the 5-year survival increase ranges from 10 to 25%. In addition to surgical resection, there are adjuvant chemotherapy schemes, such as FOLFIRINOX (a mix of Irinotecan, oxaliplatin, 5-Fluorouraci and leucovorin) or gemcitabine-based treatment. These last two drugs have been compared in the NAPOLI-3 clinical trial, and the NALIRIFOX arm was found to have a higher overall survival (OS) (11.1 months vs. 9.2 months). Despite these exciting improvements, PDAC still has no effective treatment. An interesting approach would be to drive ferroptosis in PDAC cells. A non-apoptotic reactive oxygen species (ROS)-dependent cell death, ferroptosis was first described by Dixon et al. in 2012. ROS are constantly produced in the tumor cell due to high cell metabolism, which is even higher when exposed to chemotherapy. Tumor cells have detoxifying mechanisms, such as Mn-SOD or the GSH-GPX system. However, when a threshold of ROS is exceeded in the tumor cell, the cell’s antioxidant systems are overwhelmed, resulting in lipid peroxidation and, ultimately, ferroptosis. In this review, we point out ferroptosis as an approach to consider in PDAC and propose that altering the cellular ROS balance by combining oxidizing agents or with inhibitors of the main cellular detoxifiers triggers ferroptosis in PDAC.

## 1. Introduction

Pancreatic ductal adenocarcinoma (PDAC) is the most common type of pancreatic cancer and the third cause of cancer death in both sexes. PDAC has almost the same number of diagnoses (496,000) as deaths (466,000), making it the most lethal type of cancer [1]. To date, surgical resection is the preferred method to overcome PDAC. On the one hand, tumors originating in the Vater ampulla, bile duct, or head of the pancreas are managed with a pancreatoduodenectomy (Whipple procedure). On the other hand, tumors located in the tail or body of the pancreas are surgically removed by a pancreatectomy performed distally and some cases require a total pancreatectomy [2]. Regrettably, despite the options for surgical resection, the 5-year survival rate is 25%. Moreover, this rate remains at 10% when there are no surgical resection options [3,4,5].

PDAC has a characteristic mutational profile, where mutations in the genes *KRAS*, cyclin-dependent kinase inhibitor 2A (*CDKN2A*), tumor protein p53 (*TP53*) and SMAD family member 4 (*SMAD4*) stand out [6,7]. The identification of germline mutations associated with PDAC may have major implications for treatment and familiar genetic screening. Up to 20% of patients could harbor germline/somatic mutations in genes associated with DNA damage repair (*DDR*), such as *BRCA1*, *BRCA2*, PALB2, CHECK2 and *ATM* [6]. Pathogenic somatic or germline mutations in *BRCA1/2* lead to homologous recombination repair deficit and increase the sensitivity to agents that generate crosslinks in DNA strands, such as platinum bases therapies [8].

Accumulating molecular data allows the distinction of PDAC according to molecular subtype. Bailey and colleagues differentiated four molecular subtypes of PDAC, which could lead to an improvement in the accuracy of possible future treatment [9].

In routine clinical practice, patients are categorized into three groups according to their prognosis: patients with resectable, borderline resectable or metastatic PDAC [10]. Within the resectable group, patients have to receive adjuvant chemotherapy to avoid disease relapse, which is very frequent [11,12]. Regarding the metastatic group, the most effective treatments are FOLFIRINOX and the gemcitabine plus nab-paclitaxel scheme [10]. No current data on clinical trials comparing these two treatments are available. But the effectiveness of these alternatives has been reported to be similar in a comparative study [13]. In the recent NAPOLI-3 clinical trial, the combination of liposomal irinotecan, 5-FU/leucovorin and oxaliplatin (NALIRIFOX) has improved survival of patients compared to gemcitabine plus nab-paclitaxel in OS (11.1 months vs. 9.2 months) and PFS (7.4 months vs. 5.6 months) [14].

PDAC is also characterized by an immunosuppressive tumor microenvironment (TME). Tumor-associated macrophages can raise the number of myeloid-derived suppressor cells, also known as MDSCs, which subsequently contribute to local immunosuppression [15]. Another characteristic of PDAC is that it is surrounded by a dense stroma (desmoplasia) that constricts the vasculature, limiting oxygen and drug delivery to the tumor [16,17]. The combination of the genetic profile and the hostile TME make the solid pancreatic tumor with the lowest 5-year survival rate [1]. Given the immunosuppressive environment of PDAC, restoring immunogenic capacity is a possible therapeutic target. One of these immunotherapies is against CD40, a tumor necrosis factor receptor superfamily member in macrophages, which is responsible for modulating the activation of the T-cell. Activated CD40 has been linked to the orchestration of antitumor responses [18,19,20]. Another novel approach is the development of vaccines against cancer. The GVAX vaccine consists of a whole-cell vaccine that has been transfected with the granulocyte–macrophage colony-stimulating factor acting as a maturation factor for dendritic cells. The GVAX vaccine in combination with chemoradiotherapy has been used after surgery. The vaccine showed a disease-free survival benefit of 24.8 months (95% CI, 21.2–31.6) in the GVAX group compared to 17.3 months (95% CI, 14.6–22.8) in the control group [21]. Both conventional and novel therapies are still not fully effective. Therefore, new approaches must be developed.

In this context, a new therapeutic outlook is to induce iron-dependent cell death in combination with conventional chemotherapy. Iron-dependent cell death is a type of non-apoptotic regulated cell death called ferroptosis. It is characterized by an accumulation of lipid peroxides in cell membranes [22]. Ferroptosis can occur when the cellular antioxidant systems—such as glutathione peroxidase (GPX)—glutathione (GSH)—that prevent ferroptosis are surpassed by reactive oxygen species (ROS) [23]. ROS are considered a by-product of mitochondrial metabolism, which is very active for the production of cellular ATP [24]. It is noteworthy that mitochondrial metabolism is known to play a role in susceptibility to ferroptosis through the production of ROS [25,26], which are mainly produced in the mitochondrial respiratory chain during aerobic metabolism [25,27]. Cancer cells are associated with high proliferation and active metabolism. Therefore, they need high levels of energy, resulting in an increased metabolism. This high energy demand leads to excessive ROS generation [28,29,30]. High ROS concentration has been associated with increased aggressiveness and reduced survival in the breast cancer scenario [31]. It has also been reported that PDACs with lower ROS are more resistant to chemotherapy [32], since DNA oxidation and double-strand breaks (DSBs) caused by ROS are known to result in the accumulation of multiple mutations [33]. In cancer cells, the numerous mutations increase the risk of developing resistance to conventional treatments [34].

In addition, triggering ferroptosis has been shown to be effective in overcoming chemotherapy resistance, as demonstrated by Sun et al. with ent-Kaurane diterpenoids suppressing cisplatin resistance [35]. Ferroptosis may also reverse resistance to some immunotherapies, as it has been shown that inhibition of ferroptosis promotes resistance to anti-PD-1/PD-L1 therapy [36]. In the context of overcoming resistance, ferroptosis could be responsible for re-sensitizing cells resistant to radiotherapy. Indeed, in radiotherapy, a large amount of ROS is produced by the up-regulation of ACSL4 and inactivation of ferroptosis scavengers, such as GPX4 [37].

**Figure 1:**
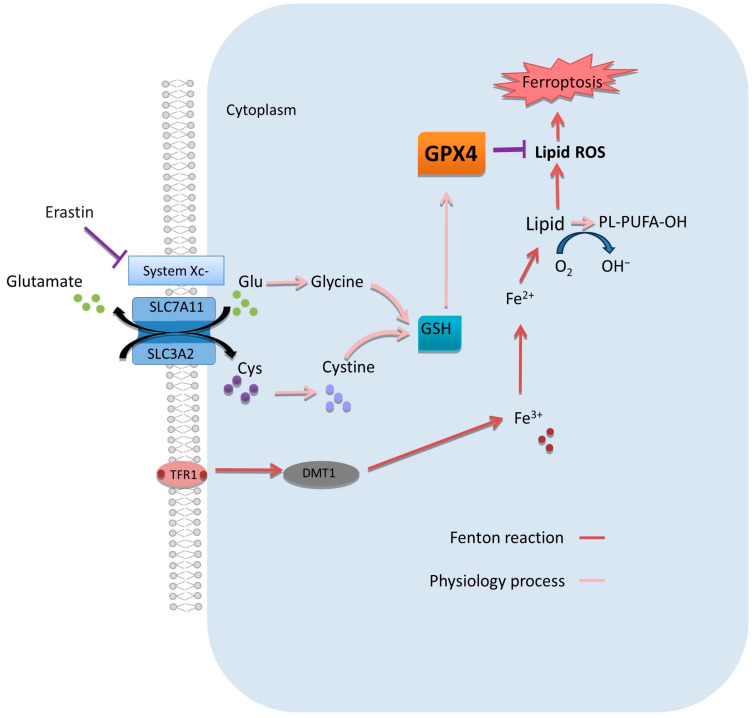
Mechanism of ferroptosis in tumor cells under physiologic and ferroptotic process. Arrows indicate activation and T bars denote repression.

Altering the ROS balance is a way to induce cell death by ferroptosis [22], which would be an effective way to kill cancer cells. Increasing intracellular ROS levels to treat PDAC is an advantage over conventional treatments as it confers chemosensitivity to cancer cells [38,39]. Hence, promoting an imbalance between iron-dependent ROS and cellular detoxification mechanisms subsequently leads to cell death by ferroptosis [23]. To disrupt the ROS balance in the cell and to cause toxicity, one possible approach would be to inhibit the GSH-GPX detoxification system. The loss of GSH would result in the loss of cellular homeostasis and ultimately cellular dysfunction causing cell death by ferroptosis (Figure 1) [40,41].

Given its therapeutic potential, here we review the possibilities of ferroptosis as a therapeutic target in PDAC due to the synergistic effect it has in combination with conventional chemotherapy.

## 2. Reactive Oxygen Species Balance in PDAC

Oxygen is an essential component of life. But it can be harmful when it causes oxidative stress. Oxidative stress is a phenomenon characterized by an imbalance between the production and detoxification of ROS [42]. These reactive species contain unpaired electrons, which makes them highly reactive oxygen free radicals. Examples of these species are superoxide (O_2_^−^) and hydroxyl (OH^−^) which can be transformed into the more stable hydrogen peroxide H_2_O_2_ [43]. ROS in the cell can act as trigger molecules for cell death, leading to ferroptosis [22].

### 2.1. Generators of ROS

**Figure 2:**
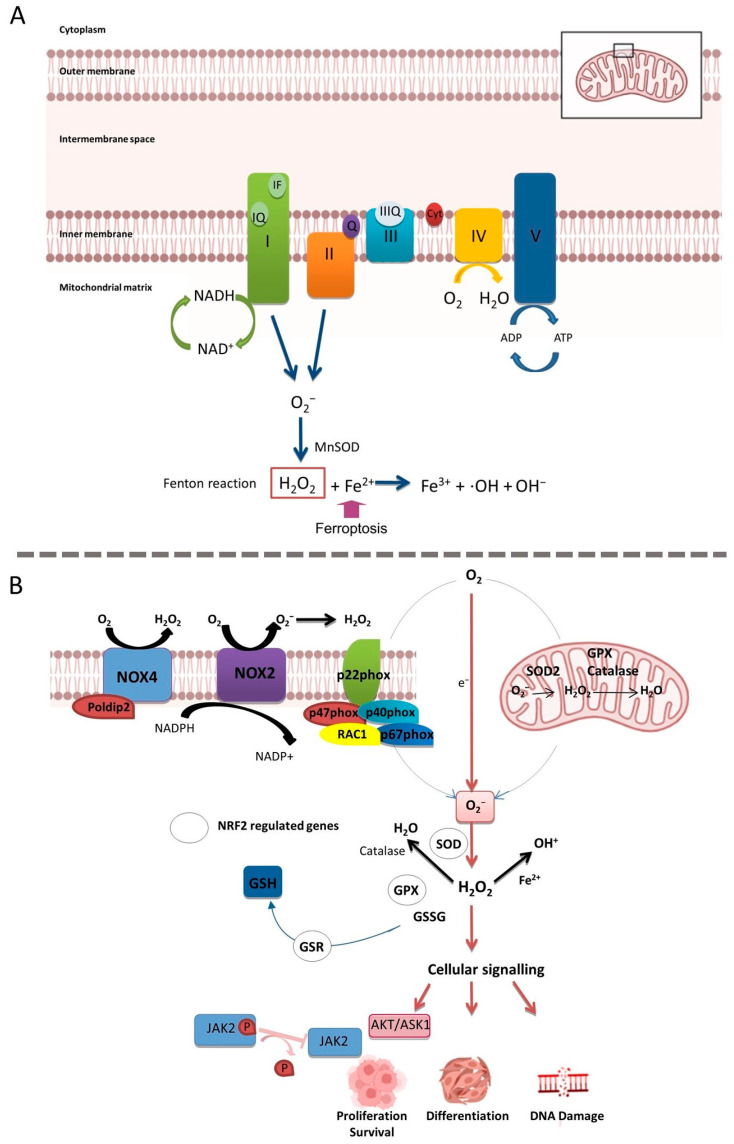
Different cellular ROS production. (**A**) ROS generation in the mitochondria. O_2_^−^ is produced in sites IQ, IF and IIIQ and converted in H_2_O_2_. Then, Fenton reaction takes place to convert H_2_O_2_ in OH^−^ and lead to oxidation of Fe^2+^ to Fe^3+^. (**B**) Intracellular ROS production in pancreatic tumor cells by NOX family of proteins and its role in PDAC maintenance. Arrows indicate activation and T bars denote repression.

ROS are continuously generated during aerobic oxygen metabolism. With approximately 80% oxygen consumption, mitochondria are the major contributor to ROS production [44]. The mitochondria are the organelle responsible for producing ATP by oxidation of lipids, glucose and amino acids. In the tricarboxylic acid cycle, an electron from these molecules is donated to the electron transport chain resulting in the oxidation of O_2_ to O_2_^−^ [45]. More specifically, O_2_^−^ is produced at several sites in the mitochondria, including complex I (sites IQ and IF) and complex III (site IIIQ). In the mitochondrial matrix, the O_2_^−^ is then converted to H_2_O_2_ by manganese superoxide dismutase (Mn-SOD). In this manner, H_2_O_2_ can be converted in OH^−^ via the Fenton reaction [25,46,47]. The Fenton reaction is another source of ROS. This catalytic reaction ends up with ferroptosis, and is dependent on ROS and ferrous ions (Fe^2+^), producing ferric ions (Fe^3+^) [48] (Figure 2A).

Additionally, ROS generated by the nicotinamide adenine dinucleotide phosphate oxidases (NADPH) family have been reported to be implicated in PDAC growth and survival (Figure 2B) [49]. These NOX-derived ROS have been reported to transmit cell survival signals via the AKT-ASK1 pathway and also by inhibiting JAK2 dephosphorylation by tyrosine phosphatases [50,51]. Within this family, there are seven members: NOX1, NOX2, NOX3, NOX4, NOX5, dual oxidase 1 (DUOX1) and DUOX2 [52]. NOX enzymes can transfer electrons throughout the membranes to produce O_2_^−^, which will be converted to H_2_O_2_ [53]. Furthermore, activating NOX can increase intracellular ROS through the interaction with Toll-like receptors, which eventually leads to the activation of the redox-sensitive nuclear factor kappa-light-chain-enhancer of activated B cells (NF-κB) and NRF2, which is a master regulator of the antioxidant response by controlling the expression of components from GSH antioxidant system (Figure 2B) [54,55]. More specifically, NOX4 is overexpressed in PDAC [56], and has been identified as a potential therapeutic target as it can generate high levels of ROS and also trigger several signaling pathways related to colorectal cancer progression [57]. This NOX-mediated ROS production has been also related to the progression of PDAC at the mRNA level [57,58].

### 2.2. Scavengers of ROS

**Figure 3:**
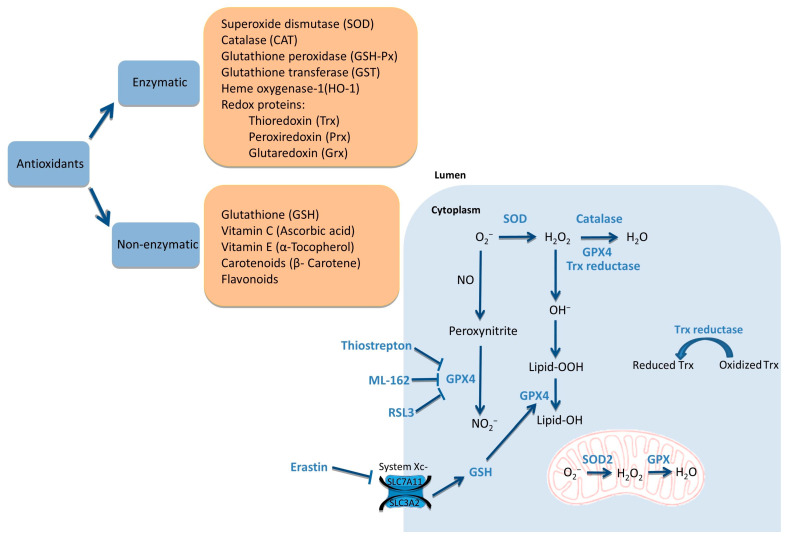
Common antioxidants and mechanism of detoxification in cytoplasm of PDAC. Arrows indicate activation and T bars denote inhibition.

From the aforementioned, high intracellular ROS levels can lead to cell death by ferroptosis. However, there are cellular control systems mainly made up of antioxidant enzymes, such as Mn-SOD, catalase (CAT), GPX and thioredoxin (TXN), and other non-enzymatic antioxidants, including GSH, ascorbic acid and tocopherol (Figure 3) [59,60,61].

Physiologically, GSH can detoxify a cancer cell in two ways: directly by interacting with ROS, or indirectly together with GPX. In the latter, GSH acts as a co-substrate of GPX in order to reduce hydrogen peroxide (H_2_O_2_) and lipid peroxide (Lipid-OOH) into water and lipid hydroxyl (Lipid-OH), respectively [62,63]. GPXs have several isoforms, of which GPX4 is known to be a central regulator of ferroptosis [23]. It has been shown that inhibition of GPX4 signaling through thiostrepton (TST) induces ferroptosis in PDAC cells (Figure 3). Likewise, RAS-selective lethal 3 (RSL3), another GPX4 inhibitor, reduced proliferation in several PDAC-derived cell lines (Figure 3) [64]. Along the same line, ML-162 acts as an inhibitor of GPX4 and induces ferroptosis in gastric cell lines (Figure 3) [65]. Similarly, erastin acts as ferroptosis inducer through the inhibition of the glutamate/cystine antiporter of system Xc^−^ and consequently suppresses the GSH synthesis [66]. These results open the door to new approaches with GPX4 inhibition as a target to increase therapeutic efficacy in combination therapies. Within mitochondrial detoxification systems, H_2_O_2_ is converted to oxygen and water by peroxidases like CAT in the cytoplasm (Figure 3) [67]. The overexpressed SOD2 system, in synergy with PPAR, inhibits mitochondrial ROS-mediated apoptosis in PDAC cell lines, which promotes PDAC proliferation [68].

### 2.3. ROS Balance

**Figure 4:**
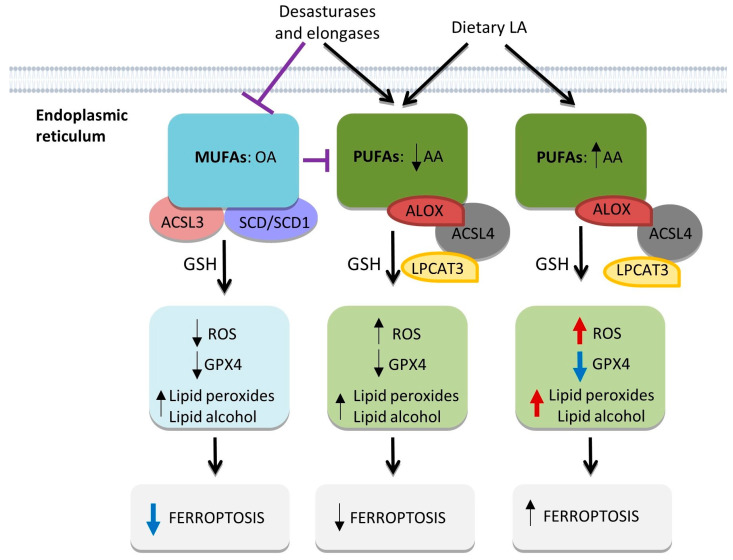
PUFA and MUFA generation, associated factors, and their involvement in ferroptosis. AA: arachidonic acid. OA: Oleic Acid. LA: Linoleic Acid. Arrows indicate activation and T bars denote repression. Blue arrows denote higher reduction and red arrows denote higher increase.

From the aforementioned research, ferroptosis is a result of missing or insufficient antioxidant defense systems, such as GPX4 and a high ROS intracellular level leading to cell death mediated by membrane lipid peroxidation (LPO) [23,69]. In particular, ferroptosis consists of iron-mediated peroxidation of polyunsaturated fatty acids (PUFAs) (Figure 4). Thus, PUFA production is mainly mediated by two enzymes—acyl-coenzyme A (acyl-CoA) synthetase long-chain family member 4 (ACSL4) and lysophosphatidylcholine acyltransferase 3 (LPCAT3) in the endoplasmic reticulum (ER)—which, in the end, causes LPO mediated by the lipoxygenase (ALOX) [70]. In fact, LPO is a process that occurs in normal cells [71], but when the GSH-GPX4 system is depleted, lipid peroxides can accumulate in cells [22,71] leading to cell membrane rupture, resulting in ferroptotic cell death. Contrary to PUFAs, monounsaturated fatty acids (MUFAs) cannot be peroxidized due to a lack of bis-allylic moieties (Figure 4) [72]. These MUFAs are synthesized by acyl-CoA synthetase long-chain family member 3 (ACSL3) or stearoyl-CoA desaturase (SCD/SCD1). Interestingly, SCD1 is overexpressed in PDAC and correlates with aggressive phenotype by increased tumor size, poor tumor differentiation and short overall survival [73]. Moreover, SCD1 together with MUFAs can compete functionally to inhibit PUFA-related ferroptosis (Figure 4) [72,74].

Considering the above, altering the ROS balance may be a key therapeutic target by inhibiting detoxification systems such as GSH-GPX4, which will thereby eliminate PDAC cells by ferroptosis.

## 3. ROS Exhibit Specific Biological Functions According to Different Molecular Subtypes of PDAC

In recent years, several cooperative networks, such as the International Cancer Genome Consortium and The Cancer Genome Atlas (TCGA) have been able to distinguish different types of tumors by molecular differences [75,76]. With this knowledge, it is possible to stratify patients according to their differential molecular profile and offer them a specific treatment option to improve their prognosis [77]. Here, we provide an integrated phenotype on the effect of ferroptosis by ROS according to different PDAC molecular subtypes. The rationale to set different molecular subtypes of PDAC is based on the need to stratify patients to perform personalized medicine.

To enhance therapeutic opportunities in PDAC, a molecular taxonomic classification was established to take advantage of the molecular subtypes of pancreatic cancer [9,78].

As mentioned above, PDAC harbors a variety of genetic mutations that lead to *KRAS* activation as well as the loss of *TP53*, *SMAD4* and *CDKN2A* [6,7,79]. In addition to these major alterations, there are other molecular mechanisms playing a role in the deregulation of cellular processes: DNA damage repair, TGF-β signaling or cell cycle regulation [78]. A better understanding of gene signaling networks may open the door to cluster PDACs with the goal of developing better therapies.

Bailey and colleagues identified four main molecular subtypes with a whole transcriptomic profile obtained by RNA-seq analysis of 266 primary PDAC samples. These subtypes were squamous (quasi-mesenchymal), pancreatic progenitor (classical), immunogenic and aberrantly differentiated endocrine-exocrine (ADEX, exocrine-like) [78]. Subsequently, Collisson et al. proposed a new stratification based on three different subtypes: quasi-mesenchymal, which is very similar to the squamous molecular subtype proposed by Bailey; the classical subtype equivalent to the previous progenitor subtype; and the exocrine-like subtype previously reported as ADEX [9]. The integration between molecular profiling, histopathology and their microenvironment has been proposed by our group. This integration can significantly help in observing ROS sensitivity in different subtypes of PDACs and could allow us to identify which ones could be more susceptible to cell death by ferroptosis [80].

### 3.1. The Squamous Molecular Subtype and ROS

**Figure 5:**
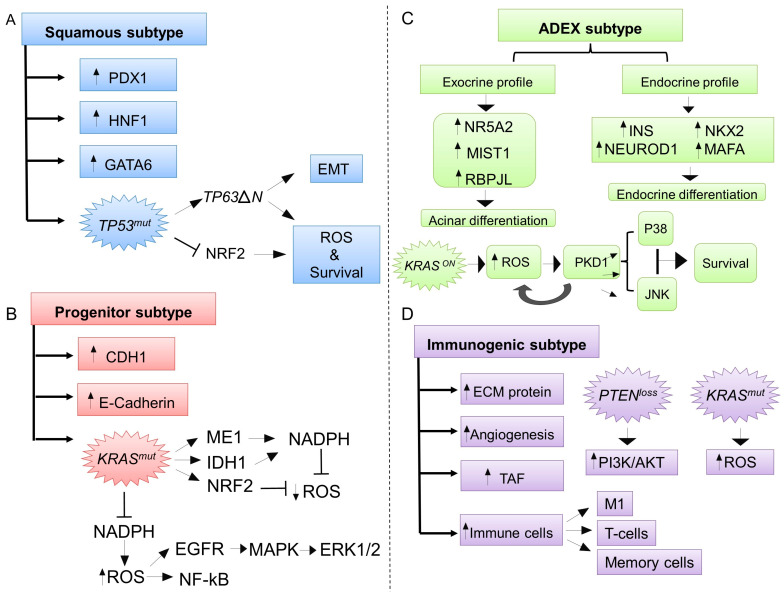
ROS involvement according to different molecular subtypes of PDAC. (**A**) ROS modulation in PDAC squamous molecular subtype and the crucial role of TP53 as an antioxidant. (**B**) ROS modulation in progenitor molecular subtype where *KRAS^mut^* plays a crucial role. (**C**) Aberrantly differentiated endocrine–exocrine (ADEX, exocrine-like) and factor involved in ROS generation. (**D**) ROS generation and molecular features of the immunogenic molecular subtype of PDAC. Arrows indicate activation and T bars denote inhibition.

The squamous subtype is characterized by a marked mesenchymal phenotype, conferred by the expression of PDX1, HNF1B and GATA6 (Figure 5A). This subtype is related to the worst prognosis due to its capacity to drive epithelial-to-mesenchymal transition (EMT), and therefore, metastases [9,81]. Moreover, the squamous subtype is enriched by TP53 mutations, and presents activation of the TP63ΔN associated network (Figure 5A). TP63ΔN underlies the development of an EMT-like program and maintains the core regulatory network responsible for chromatin accessibility, epigenetic modifications and gene expression patterns (Figure 5A) [9,82]. Normally, P53 can confer antioxidant properties to the cells, providing antitumoral protective functions [83,84]. More specifically, the Tumor Protein 53-Induced Nuclear Protein 1 (TP53INP1), a target of P53, plays a key role in antioxidant defense by participating in the elimination of ROS-producing altered mitochondria [85,86]. Moreover, the functions of the key antioxidant regulator NRF2 can be attenuated by P53 mutant proteins (Figure 5A) [87,88,89].

### 3.2. The Progenitor Molecular Subtype and ROS

The pancreatic progenitor subtype was equivalent to the classical subtype and featured the epithelial-like phenotype. It is characterized by high expression levels of epithelial and adhesion-associated genes, such as CDH1/E-cadherin [9,78]. This subtype has molecular features of *KRAS^mut^*-dependent PDAC [90]. In addition, FOXA2/3, PDX2, MNX1 and GATA6 are highly expressed in the pancreatic progenitor subtype contributing to early pancreatic development (Figure 5B) [9]. In fact, ROS are critical in the *KRAS-*driven PDAC [91]. It has been shown that O_2_^−^ is a pro-survival factor in PDAC. Consequently, oncogenic *KRAS* can increase ROS by mitochondrial dysfunction and alteration of NADPH oxidase activities (Figure 5B) [92]. Thus, the mitochondrial ROS can drive essential signaling routes, such as ERK1/2 from the MAPK signaling pathway by upregulation of epidermal growth factor receptor (EGFR) and activation of NF-κB, both of which are implicated in PDAC progression (Figure 5B) [91,93,94]. In this regard, to counteract the high ROS levels due to cancer progression, oncogenic *KRAS* can up-regulate the antioxidant defense systems. This can occur through NADPH generation by malic enzyme 1 (ME1) and isocitrate dehydrogenase 1 (IDH1), or by contrast through NRF2 activation (Figure 5B) [95,96].

### 3.3. The Aberrantly Differentiated Endocrine-Exocrine (ADEX, Exocrine-Like) Subtype and ROS

ADEX tumor subtype stands out for exhibiting transcriptional programs characteristic of a more terminally differentiated normal pancreas. This subtype can exhibit both an exocrine or an endocrine transcriptional profile [9]. On the one hand, the main networks identified in ADEX involve the transcription factors NR5A2, MIST1 and RBPJL, whose main role is to drive acinar differentiation, pancreatitis and regeneration (Figure 5C) [78,97,98]. On the other hand, INS, NEUROD1, NKX2-2 and MAFA are associated with endocrine differentiation and onset diabetes (Figure 5C) [78]. This genetic profile has been linked to *KRAS* activation [9,78]. Concerning ROS generation in this subtype, the serine/threonine kinase Protein Kinase D1 (PKD1) is a major contributor to mitochondrial ROS (mtROS) generation [99]. Indeed, when activated, PKD1 can increase PDAC cancer cells’ survival through the inactivation of c-Jun N-terminal kinases (JNK) 1/2 and P38 signaling (Figure 5C) [100].

### 3.4. The Immunogenic Subtype and ROS

The immunogenic subtype has a molecular profile very similar to the pancreatic progenitor subtype. This profile can be differentiated by the pattern of the immune infiltration resident within the TME, and more specifically, in the stroma. This stroma is comprised of extracellular matrix proteins, tumor-associated vasculature, fibroblasts and immune cells, which engender a poor prognosis in PDAC and ultimately hinder drug delivery [101]. From the foregoing, this subtype is associated with a decrease in tumor-like cells, and consequently, immune cells play a major role in this tumor subtype [9]. Thus, in tumors where there is a significant infiltration of immune cells, such as M1-like macrophages, there is a higher survival rate due to greater activation of resident memory cells (Figure 5D) [102]. Nevertheless, this immunogenic PDAC is associated with the classical subtype (previously described by Collisson et al.), which was, in turn, governed by the oncogenic phenotype of *KRAS* and the oxidative stress it caused conferring a pro-survival factor to PDAC cells [9,92,95,96]. The oncogenic phenotype of activated *KRAS* is followed by loss of phosphatase and tensin homolog (*PTEN*), which is a tumor suppressor gene that inhibits the PI3K/AKT signaling pathway and has a prevalence of 60% in PDAC (Figure 5D) [103].

Considered together, these molecular scenarios bring new rationales for different possibilities for inducing cell death in PDAC. Thus, recapping the aforementioned, ferroptosis is a type of cell death that has a lot of potential [22,104]. If we can take into account the molecular subtypes of PDAC, we can enhance its effectiveness, and provide the best treatment for each patient to solve a personalized medicine challenge. Following this assumption, all pancreatic progenitor, ADEX and immunogenic patients could be treated in order to increase intracellular ROS and decrease the levels of antioxidant systems, as previously described with oncogenic *KRAS* colorectal cancer (CRC) models treated with β-elemene (*Pterodon emarginatus*) and Cetuximab [105]. Also, in these three PDAC subtypes, it could be interesting to target the NRF2 signaling pathway. Cetuximab is reported to boost RSL3-induced ferroptosis by inhibiting the NRF2 pathway in *KRAS*-mutated CRC cells [106]. Regarding squamous PDAC, it presents a mutated TP53 genetic background, which offers a divergent model of ROS production and hence ferroptosis [107]. On the one hand, P53 can decrease intracellular levels of ROS and regulate metabolic intermediates, such as TIGAR and GLS2, that decrease cellular levels of ROS [108,109]. On the other hand, P53 has been found to play a major role in inducing ferroptosis by transcriptional repression of SLC7A11, impairing cysteine import and promoting ferroptosis initiation [107,110]. Therefore, new approaches to studying the role of P53 in ferroptosis are needed.

In that sense, ferroptosis is the final process to which all of these pathways would lead. Similarly, the activation of caspases by macromolecular processes results in the activation of procaspases and the proteolytic process that will lead to cell death by apoptosis [111]. Meanwhile, ferroptosis is a caspase-independent cell death that is produced by PUFA oxidation via intracellular high ROS levels [112]. Lipoxygenases (LOX) activity can catalyze PUFA-containing phospholipids and generate a pool of pro-ferroptotic peroxidized lipids [113]. Subsequently, iron is an essential component of the Fenton reaction to produce an intracellular ROS [114]. Free iron in the cell is essential for ferroptosis. However, iron is not required to trigger apoptosis. Iron chelators can act as control systems for the ferroptosis [115,116]. In this regard, GPX4 may act by preventing the toxicity produced by peroxidized lipids while maintaining the integrity of the lipid bilayer [117].

Given this overview of PDAC molecular subtypes and their involvement in the ferroptosis process, we will now analyze the interaction between ROS and chemotherapy treatments commonly used in routine clinical practice.

## 4. Reactive Oxygen Species and Commonly Used Chemotherapy in PDAC

From the aforementioned, PDAC can only be resected in a minority of cases, and the prognosis for survival after surgery is about one year [118,119,120]. At this point, combined chemotherapy is the most used option to treat PDAC patients after resection and in unresectable cases to improve disease-free survival and overall survival [121]. ROS production is a shared consequence among chemotherapies due to their involvement in triggering cell death. Therefore, combinatorial therapy based on ROS generation to increase toxicity and susceptibility to other adjuvant therapies would be a potential therapeutic approach. In this section, the modulation of ROS by the main chemotherapeutics against PDAC will be discussed.

**Figure 6:**
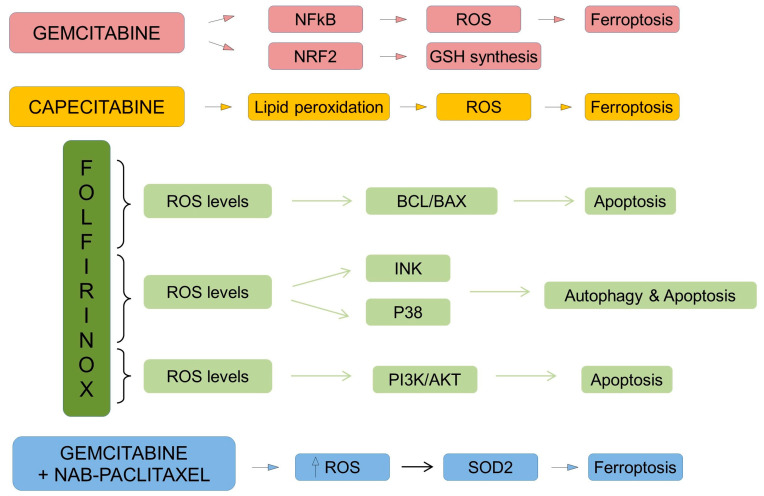
ROS modulation by most used chemotherapies against PDAC. Nab-Paclitaxel: nano-albumin bound paclitaxel.

Gemcitabine monotherapy has been traditionally the cornerstone of chemotherapy in PDAC. Now, it is used in frail patients. It is known that gemcitabine induces the accumulation of ROS during treatment, which is a new cytotoxic effect that has come under the spotlight in recent years [122]. Some studies have shown mechanisms by which gemcitabine can cause ROS accumulation through NF-kB activation [123], and thus have a possible role in favoring ferroptosis. It has also been reported that gemcitabine can induce GSH synthesis by activation of NRF2. Thus, favoring gemcitabine resistance in the end (Figure 6) [124].

While less recommended, capecitabine monotherapy is also useful in selected patients and in combination with gemcitabine [10]. A study using cardiomyocytes reported that capecitabine could cause lipid peroxidation and oxidative stress [125]. Thus, it causes ferroptosis, thereby increasing its cytotoxic effect.

Within polychemotherapies, FOLFIRINOX is used in the first line of treatment for PDAC [10]. This chemotherapeutic regimen contains 5-FU, which has been shown to elevate ROS levels and trigger mitochondrial dysfunction by up-regulating BAX/BCL-2. In a study, it was found that suppressing the generation of ROS abolished the cell growth inhibition of 5-FU [126]. Irinotecan has been reported to increase intracellular ROS levels and cause cell death by the activation of JNK and P38 MAPK pathways (Figure 6) [127]. Oxaliplatin can also increase intracellular ROS and cause apoptosis in a dose-dependent manner in PANC-1 and MIA PaCa-2 [128]. In combination, these drugs that make up FOLFIRINOX can have a synergistic cytotoxic effect in such a way as to generate elevated oxidative stress to ultimately cause ferroptosis.

To follow up with polychemotherapies, a common combination to treat metastatic PDAC is gemcitabine plus albumin-bound paclitaxel [10]. It has been shown that paclitaxel can increase the amount of intracellular ROS and inhibit the antioxidant action of SOD-2 (Figure 6) [129]. The combination of gemcitabine and paclitaxel can be very interesting since increasing intracellular ROS levels and inhibiting antioxidant systems can cause cell death by ferroptosis, enhancing cytotoxicity and improving the efficacy of chemotherapy.

## 5. Potential Clinical Benefits of Ferroptosis Modulation in PDAC

As previously reported, current cancer treatments have limited clinical efficacy in PDAC patients. An important reason for therapeutic failure is drug resistance, especially when cancer cells are able to avoid apoptosis [130]. In this review, we discuss how ROS and ferroptosis are a potential route to tackle cancer, specifically altering ROS balance to produce cell death through ferroptosis. This could be a chemotherapeutic option per se or enhance the effect of other treatments. In this section, the main approaches to alter ROS and produce ferroptosis in PDAC will be addressed, as well as the possibilities to be set as a new chemotherapeutic regimen alone or used in combination to enhance current adjuvant therapies.

### 5.1. Targeting Oxidants

It is known that tumor cells produce significant amounts of ROS when they grow and metastasize. Despite the high amount of ROS, tumor cells manage to maintain the redox balance. This ability is due to the maintenance of a high expression of antioxidant systems, which ultimately prevents exceeding the ROS threshold that would lead to cell death by apoptosis or ferroptosis [131,132].

**Figure 7:**
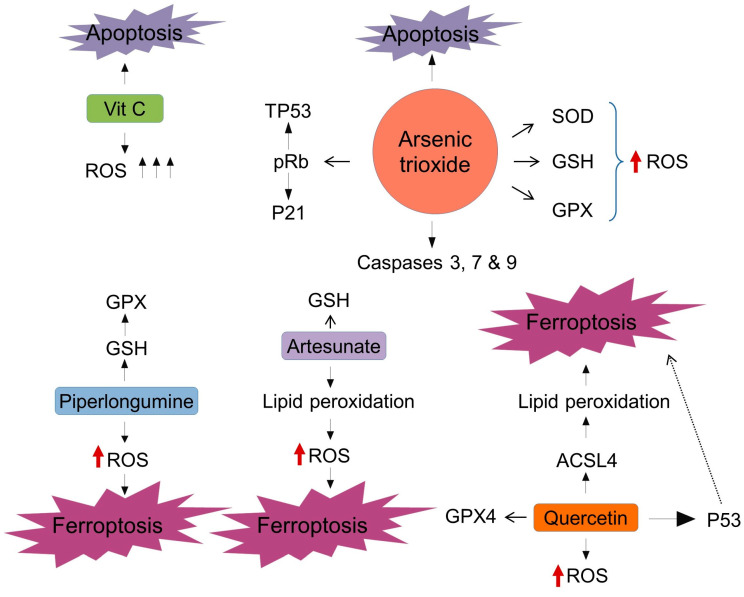
Molecular strategies to target oxidants as a new approach against PDAC.

Arsenic trioxide (ATO) is considered an oxidizing agent. It is capable of producing oxidative damage not only by inhibiting antioxidant systems, such as SOD, GSH or GPX, but also through the production of ROS (Figure 7) [133]. Regarding the production of ROS by ATO, it has been shown that its administration can cause overexpression of Caspases 3, 7 and 9 in PDAC cells, which in turn leads to apoptosis [134]. Moreover, ATO has been reported to cause cell cycle arrest at the G1 or G2-M phase as long as RB is hypophosphorylated and CDC25 B/C phosphatases are reduced by the inhibition of CDK2/6 and CDC2-associated kinases [135]. Another property of ATO is that it can restore the correct folding of TP53 proteins more efficiently than other TP53 reactivating molecules [136], which could be an interesting approach in PDAC. ATO is currently approved therapy for high-risk acute promyelocytic leukemia [137]. But its therapeutic potential for PDAC patients does not seem as promising in pre-clinical studies. For instance, phase II study did not show a significant response with a median survival of 3.8 months [138].

Furthermore, vitamin C is among the molecules that have a pro-oxidant effect. Although it has traditionally been identified as an antioxidant, high doses of vitamin C have antitumor effects by generating large amounts of intracellular ROS. This is due to the fact that tumor cells are forced to reduce a large amount of dehydroascorbate (DHA), which is an oxidized form of vitamin C (Figure 7) [139]. Additionally, among the oxidizing molecules, piperlongumine (PL) and artesunate (ART) have been proven to induce an increase in ROS causing cell death. It has been reported that in a dose-dependent manner, PL increases ROS levels intracellularly and inhibits GPX activity by GSH depletion, which ultimately induce ferroptosis (Figure 7) [140]. ART has been shown to induce iron-triggered and ROS-mediated cell death in different PDAC cell lines [141]. Along the same line, it leads to GSH depletion and lipid peroxidation and, finally, triggers ferroptosis. There are some pharmacological agents that use these pathways to induce cell death through oxidative stress, such as imidazole ketone erastin (IKE) and cyst(e)inase [142,143]. In addition, antioxidant inhibitors, such as RSL-3 and ML-162 are used to induce oxidative stress. The mechanism of action of these inhibitors involves the inhibition of GPXs. These inhibitors have been shown to increase lipid peroxidation and cause ferroptosis in PDAC [144] and in head and neck cancer-derived cell lines [65]. In another study carried out in PDAC cell lines, laminarin, a glucan derived from brown algae, has been tested as a possible inducer of cell death. In the study, they found that laminarin could increase ROS levels, trigger apoptosis and inhibit cell proliferation and migration in a dose-dependent manner [145].

### 5.2. Targeting Antioxidants

In PDAC development, tumor cells produce large amounts of ROS and other free radicals that help tumor cells grow, survive and proliferate [92]. ROS have been linked to the initiation of tumorigenesis and the alteration of cellular processes through their effect on protein function [146]. Hence, an excessive amount of oxidative stress causes the total loss of function of some proteins, which are necessary for the adaptation of metabolic and antioxidant programs involved in the clearance of oxidative stress [147].

Therefore, maintaining the balance of antioxidants is important as there are all types of enzymes or cofactors that are involved in the elimination of intracellular ROS. There are two classes of antioxidants: endogenous and exogenous antioxidants. Among endogenous antioxidants, the main cellular antioxidant system is GSH-dependent in which GSH is used as a cofactor by GSH S-transferases (GSTs) and GSH peroxidases (GPXs) to eliminate ROS. Besides the GSH-GPXs axis, there are networks based on peroxiredoxins (PRDXs) regeneration, which are sulfaredoxin (SRX) and thioredoxin. This enzyme cluster exhibits high catalytic activity toward H_2_O_2_ [23,148]. Furthermore, the antioxidant systems SOD1 and SOD2 stand out and act at the cytosol and mitochondrial level, respectively. They play a role in the ROS scavenging [60]. It has been shown that the loss of antioxidants, such as GPX1, GPX3 or SOD2, could increase tumor progression in several mouse models [149,150]. In addition, in a mouse model of PDAC, metastasis was decreased by injection of TIGAR, an endogenous antioxidant [151].

Many exogenous antioxidants have been evaluated in in vitro models and clinical trials to test their antitumor efficacy. The rationale for this is based on several studies that have reported that certain antioxidants ingested in the diet can inhibit tumor cell proliferation, and even cause tumor cell apoptosis, particularly in combination with chemotherapy [152]. Several flavonoid compounds, such as genistein, capsaicin and benzyl isothiocyanate, which are derived from plants, have been tested for their ability to induce apoptosis in PDAC cell lines [153]. In addition, studies have been reported with δ-Tocotrienol, a natural form of vitamin E, which acts as an antioxidant. This compound has been shown to inhibit tumor growth and metastasis in PDAC in mice models [154]. Empowering antioxidant systems is an interesting strategy in PDAC to decrease DNA damage caused by oxidative stress and decrease tumor growth potential.

### 5.3. Combination Approaches

Considering oxidizing agents and antioxidants separately, we have been able to see that certain compounds exhibit antitumor activity on their own. But the most interesting approach would be a combination of oxidants or antioxidants together with chemotherapy schemes. The rationale of these combinations would be to sensitize PDACs to chemotherapies by modulating oxidant and antioxidant compounds since resistance to chemotherapy is still a limiting factor in PDACs.

Among the possible combinations of therapeutic agents, stress-inducing agents stand out. These agents include the combination of etoposide and trigonelline in in vitro and in vivo models of PDAC. Trigonelline is a coffee alkaloid that has been shown to cause lipid oxidation and susceptibility to apoptosis. In combination with the topoisomerase II inhibitor agent, etoposide potentiates apoptosis in PDAC models [155]. Moreover, a study has reported that the diterpenoid libertellenone H (LH) is able to inhibit the antioxidant system Thioredoxin and induce ROS-mediated apoptosis in PDAC cell lines. It has also been reported that this effect could be reversed by the Mn-SOD enzyme [156]. So, an interesting approach would be to combine LH with an inhibitor of the antioxidant Mn-SOD to enhance the oxidative effect that triggers apoptosis.

In line with the combination of oxidizing agents and chemotherapy, a possible combination of oxaliplatin with a mangiferin, a glucosylxanthone, aims to be effective in inducing cell death in PDAC. On the one hand, it has been shown in a study with PDAC cell lines that mangiferin can induce autophagy, mitochondria-triggered apoptosis, cell cycle arrest and the suppression of tumor migration and invasion [157]. On the other hand, in a previous study, it has been shown that the combination of mangiferin and oxaliplatin has a synergistic effect favoring apoptosis and opens the door to the reduction in resistance to chemotherapy in different cancer cell lines [158]. Meanwhile, some widespread flavonoids, such as quercetin can produce P-53-independent ferroptosis in cancer cell lines. Quercetin can increase the amount of intracellular and mitochondrial ROS, as well as increase the production of ACSL4, which ultimately alters the balance towards ferroptosis through lipid peroxidation. Contrary to this, it was observed that in quercetin-treated cells, GPX4 expression was decreased, further altering the balance towards ferroptosis (Figure 7) [159].

This approach could be interesting in PDAC since it is a pathway that induces cell death by ferroptosis and is independent of P53. So, it would be even more interesting to investigate it in the squamous–P53 deficient PDAC. To support combinatorial therapies between oxidative compounds, it has been shown that the combination of cotylenin A (CN-A) and phenethyl isothiocyanate (PEITC) can produce ROS-triggered ferroptosis in PDAC cells. This joint effect of CN-A and PEITC could be reversed with a ROS scavenger (N-acetylcysteine) together to a ferroptosis inhibitor (liproxstatin). Thus, demonstrating the ROS-enhancing effect of this combination of compounds to produce ferroptosis [160]. In line with this, a previous study reported that CN-A showed a synergistic effect on cell death and reduced invasiveness in breast cancer cells [161].

In addition to the approaches mentioned above, there are other ways to modulate ferroptosis. Among these is the regulation of molecules essential in the maintenance of cellular energy balance, such as AMPK. The role of AMPK in ferroptosis resistance was evaluated in ferroptosis-resistant kidney cell lines (ACHN). This cell line, also characterized by high basal AMPK expression, was subjected to treatment with compound C, resulting in ferroptosis sensitization of cells that were resistant when treated with erastin or cisterna detection [162].

## 6. Conclusions

In conclusion, all these different approaches on how to alter the ROS balance to trigger ferroptosis directly or through the intermediates involved turn out to have great potential in PDAC, a type of tumor with a poor prognosis where current treatments are not fully effective. In this review, the possibilities of modulating ferroptosis in the PDAC have been explored. Numerous ferroptosis activators are currently under development, with a particular focus on GPX4. However, our approach goes far beyond inhibiting GPX4 and proposes different alternatives to cause the increase in ROS that triggers lipid peroxidation and thus ferroptosis. The main idea and future perspectives will focus on therapy based on combinations of oxidizing agents, to increase ROS and limit the negative effects of each oxidant separately. Consequently, considering these combinatorial therapies targeting ROS-dependent ferroptosis, the therapeutic options in the clinic would increase and with them the outcome of PDAC patients.
